# DCNet: Densely Connected Deep Convolutional Encoder–Decoder Network for Nasopharyngeal Carcinoma Segmentation

**DOI:** 10.3390/s21237877

**Published:** 2021-11-26

**Authors:** Yang Li, Guanghui Han, Xiujian Liu

**Affiliations:** 1 School of Mathematics, Sun Yat-sen University, Guangzhou 510275, China; liyang259@mail2.sysu.edu.cn; 2 School of Biomedical Engineering, Sun Yat-sen University, Shenzhen 518107, China; hangh3@mail.sysu.edu.cn; 3 School of Information Engineering, North China University of Water Resources and Electric Power, Zhengzhou 450046, China

**Keywords:** DenseNet, supervised learning, nasopharyngeal carcinoma segmentation

## Abstract

Nasopharyngeal Carcinoma segmentation in magnetic resonance imagery (MRI) is vital to radiotherapy. Exact dose delivery hinges on an accurate delineation of the gross tumor volume (GTV). However, the large-scale variation in tumor volume is intractable, and the performance of current models is mostly unsatisfactory with indistinguishable and blurred boundaries of segmentation results of tiny tumor volume. To address the problem, we propose a densely connected deep convolutional network consisting of an encoder network and a corresponding decoder network, which extracts high-level semantic features from different levels and uses low-level spatial features concurrently to obtain fine-grained segmented masks. Skip-connection architecture is involved and modified to propagate spatial information to the decoder network. Preliminary experiments are conducted on 30 patients. Experimental results show our model outperforms all baseline models, with improvements of 4.17%. An ablation study is performed, and the effectiveness of the novel loss function is validated.

## 1. Introduction

Nasopharyngeal Carcinoma (NPC) is a type of malignant tumor with high incidence in East and Southeast Asia. In 2018, about 129,000 cases were reported, and more than 70% of the new cases were diagnosed in the region mentioned. Since Nasopharyngeal Carcinoma is sensitive to ionizing radiation, the treatment of Nasopharyngeal Carcinoma is mainly based on radiotherapy [1]. There are three stages of radiotherapy technique improvement: conventional 2D, 3D conformal, and then intensity-modulated. Presently, intensity-modulated radiotherapy (IMRT) is widely used and has reduced the 5-year occurrence rates of locoregional failure for newly diagnosed and non-metastatic Nasopharyngeal Carcinoma to 7.4% [2,3]. Despite many significant radiotherapy technique improvements, precise delineation of the gross tumor volume (GTV) plays an important role in all techniques proposed [1]. IMRT is an advanced type of high-precision radiotherapy that uses a computer-controlled linear accelerator to deliver modulated radiation doses to malignant tumors. A precise 3D segmentation of the malignant tumor is needed to determine the dose intensity pattern conforming to the tumor shape best to maximize tumor dose while also minimizing the dose to adjacent normal tissues. Hence, dose delivery accuracy hinges on the precision of the delineation of the GTV of the tumor. However, manually delineating the GTV is labor-intensive and time-consuming since manual segmentation of a brain or neck tumor typically costs 2.7 h [4]. Moreover, inter-observer variability always remains high due to variations in oncologists’ experience. Therefore, methods of automatic GTV segmentation are in demand to relieve oncologists from heavy work and elevate the consistency and accuracy.

**Figure 1:**
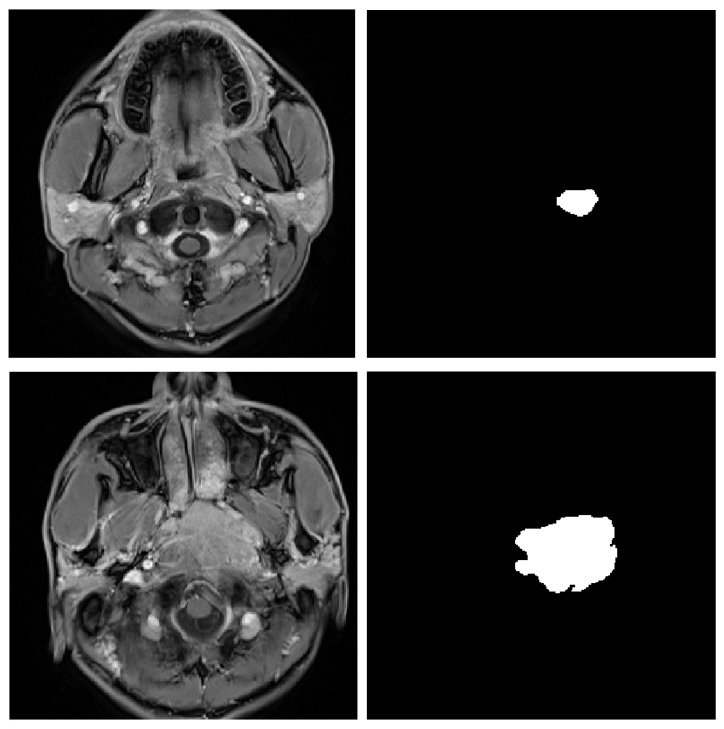
A sample of the significant variation of the scales of tumors. Most current methods are not able to perform consistently precisely on such slices with noticeable different scales.

Although some methods for automatic GTV segmentation have been proposed (See Section 2), a problem of significant intraclass variation remains not solved for medical image segmentation. Therefore, we are inspired by [5] and propose a densely connected deep convolutional encoder–decoder network for Nasopharyngeal Carcinoma segmentation. A tumor is a lump composed of Carcinoma cells, usually, with no particular shape instead of a regular cylindrical shape [6]. Hence, when it comes to Nasopharyngeal Carcinoma segmentation, the areas of segmented masks always vary depending on anterior coordinates of the medical image slices (See in Figure 1). Therefore, using multi-scale semantics in the feature extraction (encoding) stage is important for refining segmentation results. U-Net [7] extracts multi-scale semantics through skip-connection architecture. It is simply a concatenation of feature maps in the encoding stage and maps generated in the corresponding decoding (up-sampling) process. However, we are unable to bring some optimality or rationality to the concatenation operator. So, we assume that some kinds of operators acting on feature maps in the encoding stage before concatenation can obtain better results. In addition, convolution approximates the optimal operator. Additionally, to expand the receptive fields, a Pyramids Pooling Module (PPM) [8] is added. Since too many layers may cause the gradient vanishing problem during training, we modify supervised loss via integrating loss functions plugged at every feature fusion level. The novel loss function also contributes to the ability of the model to achieve an equal performance of multi-scale tumor regions detection because outputs from all decoding levels of various scales are fused and leveraged. The innovations of the work are summarized as follows:

1. We proposed a deep densely connected convolutional network with a pyramid pooling module followed to establish a multi-level representation of multi-scale feature maps. The size of GTV probably changes dramatically as the z-axis of the transverse slice changes. To remedy the large-scale variation, we deploy five densely connected convolutional blocks with expansive receptive fields to detect multi-scale regions of tumors and leverage a pyramid pooling module to fuse the information from different levels. The high-level feature maps with larger receptive fields contribute to locating tumors, and the low-level feature maps with smaller receptive fields with more spatial information contribute to refining segmentation.
2. We involved and modified skip-connection architecture to our network and brought three-fold benefits: the relatively low-level spatial information is propagated to the corresponding level in the decoder network; the feature maps from the decoder network are convoluted before concatenation which outperforms direct concatenation; the convolutional layers added to compress the channels of feature maps to reduce the number of parameters and accelerate training steps.
3. The multi-scale and multi-level loss functions contribute to realizing multi-scale supervision. It enhances the performance of multi-scale GTV detection.

The remainder of this paper is organized as follows. We give a review of related work in Section 2. In Section 3 we introduce the proposed DCNet in detail. Section 4 describes our experimental setup, performance metrics, and discusses experimental results. Finally, the conclusions and future work are laid out in Section 5.

## 2. Related Work

Previous studies on Nasopharyngeal Carcinoma segmentation are roughly categorized into traditional methods and deep learning methods, which are to be introduced, respectively.

### 2.1. Traditional Methods

Traditional visual segmentation algorithms before learning-based methods always extract image features manually and classify voxels or pixels based on extracted features, which are perceived as semi-automatic methods. Fitton et al. [9] proposed an operator-guided delineation tool to refine physician-drawn contours of the GTV assisted by a snake algorithm using defined attraction force as an image feature. Because of demands for automatic segmentation methods, machine learning is prevalent and widely used in clustering and classification tasks. As segmentation is equal to a pixel/voxel-wise classification or clustering task, many learning-based methods have been applied to NPC segmentation. Since support vector machine (SVM) [10] is a typical learning-based classifier, it is widely used. Zhou et al. [11] presented a two-class SVM based method. SVM can learn the actual distribution of magnetic resonance (MR) image data without prior knowledge, and segmentations are performed by a trained SVM classifier. Huang et al. [12] introduced two region-based methods with parameters learning. One is based on metric learning by defining a spatially weighted metric-based similarity function, and the other is based on SVM. Moreover, some probabilistic graphical models are also exploited. Tatanun et al. [13] employed a probabilistic model to select potential seeds for region growing process subsequently. Huang et al. [14] proposed a hidden Markov random field model with maximum entropy to model the spatial information with prior knowledge and achieved good results. Despite precise segmentation obtained from specialized datasets, most conventional machine learning methods require handcrafted feature extraction from the datasets to be trained and their consistency in heterogeneous datasets, where considerable variation in medical image modality (CT/MR), image position (patient), spacing, etc., probably exists. Hence their generalization ability cannot be ensured.

### 2.2. Deep Learning Methods

In recent years, many deep learning methods have been applied to medical image segmentation and outperform many conventional methods such as level set [15], watershed [16], and K-means [17]. Since convolutional neural networks (CNN) [18] has proved to be a precise and efficient method of semantic image segmentation since it is end-to-end and able to capture contextual semantics through computing high-level feature maps, SegNet [19] referring to a fully convolutional network (FCN) [20] as the encoder stage was first proposed. Then Olaf Ronneberger proposed U-Net [7], one of the most successful networks based on FCN structure, which has been a baseline network architecture in medical image segmentation due to its powerful effect and few training parameters [21]. Many of other network architectures are its modifications such as 3D U-Net [22], V-Net [23], U-Net 3+ [24] etc.

The excellent performance of CNNs and U-Net has prompted some studies on applying them to NPC segmentation. Most investigations are innovations in network architectures, and the modifications are categorized into decoder components, input layers, and connection architecture. In terms of decoding stages, Men et al. [25] replaces up-sampling-convolution layers with deconvolution layers. The extension of input layers also is considered by some investigators. 3D U-Net [22] takes 3D medical volume data instead of 2D slices as inputs to generate dense volumetric segmentation of medical images. Beyond expanding dimensions, multi-modality fusion networks also have been experimented with. MMFNet [26] takes 3D images from three MR modalities as inputs to capture cross-modality and modality-specific features. Zhao et al. [27] invented and auxiliary paths on dual-modality PET-CT images, which takes PET and CT slices as inputs. Additionally, multi-view learning is also supposed to be a potential way [28] for biomedical image segmentation. In addition, Ma et al. [29] proposed three CNN networks taking a single view as input, respectively. As mentioned, concatenation introduced as “skip-connection” to U-Net may not be the best choice for propagating original information to a high-level feature map. Therefore, Lin et al. [30] adopts residual block to deliver context information to higher resolution layers. In addition to a conventional CNN structure, attention mechanism has been investigated in recent years, and experiments prove it is promising for improvements of the performance of CNN. CA-Net [31] involves attention mechanism by adding a spatial attention module, a novel channel attention module, and a scale attention module.

## 3. Methodology

In this section, we provide details of our DCNet in terms of the preprocessing method, the architecture of the network, and the integrated loss function. In the section about preprocessing, we mainly introduce data augmentation. Then in the next section, we present the architecture of DCNet, focusing on Dense Block, Average Pooling layer, and the skip-connection architecture. Finally, we propose an integrated loss function for multi-level supervision learning.

### 3.1. Preprocessing

The voxel values of MR scans are affected by the configuration and many complicated external factors, which consistently show high variability between scans. Hence normalization is performed initially to let all the values fall within the fixed interval [0, 1].

Since few transverse slices are labeled, data augmentation has been applied to generate training data from original slices to alleviate over-fitting. Augmentation methods include random rotation from −10 to 10 degrees, flipping, and translation. The proportion of each method accords with the work from Hao et al. [32].

### 3.2. Network Architecture

**Figure 2:**
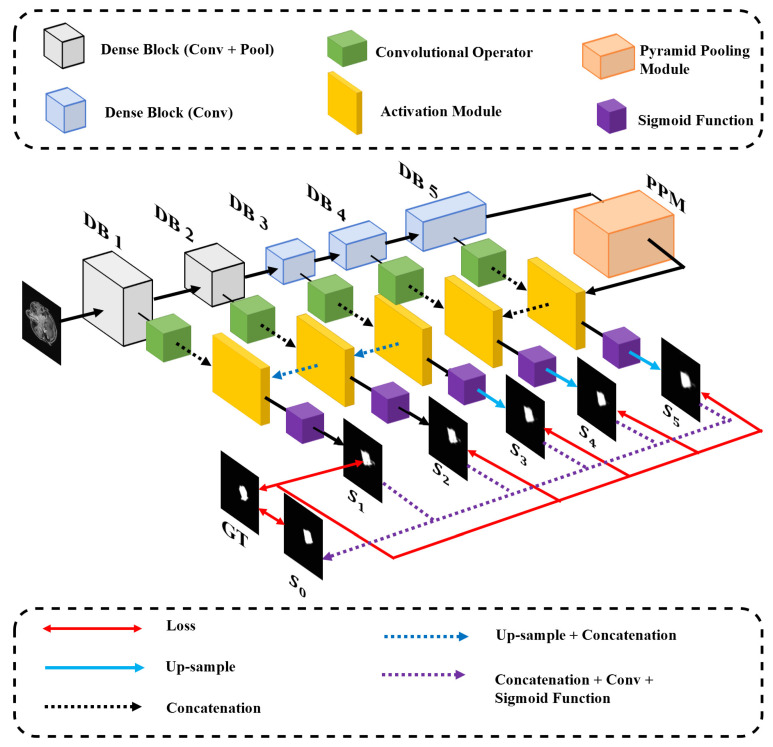
The architecture of DCNet. The growth rate [33] and the number of convolution layers in each dense block (DB) are both set to 12. The Dense Block (Conv+Pool) refers to a Dense Block (see Section Dense Block), of which the transition layer is a combination of an Average Pooling layer and a convolutional layer.

The proposed model is inspired by [5], since it performs well in locating salient objects and detecting the edges of them. They parallel the two main tasks (location of tumor regions and boundary-awareness) of Nasopharyngeal Carcinoma segmentation. The whole model is composed of the encoder network and the decoder network. The architecture of the proposed model is shown in Figure 2. In the encoder network, densely convolutional blocks (referred to as DB) are designed to extract high-level semantic information (i.e., locate tumors in MR slices). Pyramid Pooling Module (PPM) introduced is intended to represent the highest-level semantic feature further and render global contextual information in the largest receptive field to locate tumor signals roughly easier. The encoder network contains five Dense Blocks (DBs), the architecture of which is similar to the dense layer in work from Huang et al. [33]. Each DB has 12 convolutional layers, each of which is composed of a BN+ReLU+Conv [34] layer. BN+ReLU+Conv is a layer consisting of a Batch Normalization layer, a ReLU, and a convolutional operator. A BN+ReLU+Conv layer is placed between every two DBs (referred to as transition layer in DenseNet [33]) to compress the channels of the outputs of DBs. To expand receptive fields by down-sampling, a 2×2 average pooling layer is placed after the first two DBs. The numbers of the channels of the feature maps generated by DB1, DB2, DB3, DB4, and DB5 are 80, 112, 118, 136, and 16, respectively. The sizes of them are 256, 128, 64, 64, and 64, respectively. The architecture of the pyramid pooling module (PPM) is the same as the pyramid pooling module in PSPNet [8]. Feature maps with the size of 64×64 are down-sampled to four levels and then recovered to 64×64. The decoder network containing up-sampling is aimed to recover the feature maps to the size of 256×256 at different levels ($S_{0}$∼$S_{5}$) Skip-connection architecture is involved and modified to restore the low-level spatial information and propagate it to the decoder network for segmentation refinement since low-level spatial information contributes to detecting the edge of tumors. In the decoder network, feature maps are up-sampled to the same resolution as input images 256×256. $S_{1}$∼$S_{5}$ are the five feature maps produced at different levels, concatenated to generate the final segmentation $S_{0}$. The Activation Module is a BN+ReLU+Conv layer. In addition, $S_{0}$∼$S_{5}$ have corresponding loss functions $J_{0}$∼$J_{5}$ as mentioned.

#### 3.2.1. The Encoder Network

The encoder network includes the following three types of main components: Dense Blocks (DBs) is employed to extract features from input images; BN+ReLU+Conv, consisting of a Batch Normalization layer, a Rectified Linear Unit, and a convolution operator, is involved as a processing sequence to compress the channels of the outputs of DBs; Average Pooling Module is embedded to enlarge the receptive fields of subsequent Dense Blocks, through which global semantic (or contextual) feature be extracted to locate tumors. Additionally, it downsizes the training parameters.

##### Dense Block

**Figure 3:**
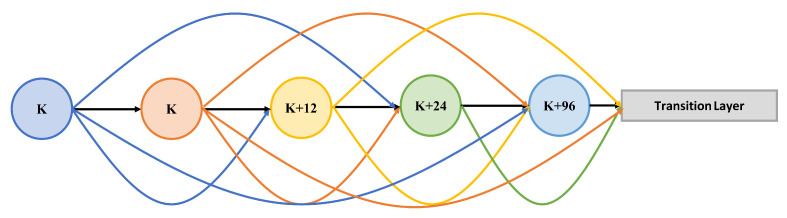
A four-layer Dense Block. The growth rate is 12. The node is a combination of a BN layer, a ReLU, and a convolutional operator. *K* means the node takes *k* inputs. The transition layer depends on the specific DB. In DCNet, the transition layers of the first two DBs (DB1 and DB2) are a combination of a convolutional operator and an average pooling layer, and the transition layers of the other DBs (DB3, DB4, and DB5) are a convolutional operator.

Densely connected convolutional network, referred to as Dense Block, is inspired by DenseNet [33]. The number of convolutional layer and feature maps each convolutional layer producing (The growth rate [33]) are both 12, The detailed architecture is shown in Figure 3. Each convolutional layer is a combination of Batch Normalization, Rectified Linear Unit (ReLU), and a convolutional operator. The Batch Normalization layer introduced is intended to remedy Internal Covariate Shift by fixing the distribution of the inputs of the ReLU [34]. As a modification, we substitute dilated convolution for general convolution for the extension of receptive fields, which is the same as [5]. Since dilated convolution expands the receptive field without increasing the number of operations [35]. At the same time, the sizes of feature maps are invariant to preserve spatial information. The feature maps produced by DBs are designed to be compressed to 16 and propagated to the decoder network as low-level (spatial detailed) information via skip-connection architecture (see Section Skip-Connection). The forward model of the kth layer in the DB can be formulated as:

(1) $$y_{k}=H\left(y_{1}⊕y_{2}⊕\ldots ⊕y_{k-1}\right)$$

where $y_{k}$ is input and output of kth layer, respectively. $y_{1}⊕y_{2}⊕\ldots ⊕y_{k-1}$ represents the concatenation in channel dimension, and the convolutional layer (more specifically, it is a combination of a Batch Normalization layer, a ReLU, and a convolutional layer) is denoted by H(). Additionally, *x* is padded with zeros to preserve the size of feature maps. (referred to as same padding in [36]). Outside the boundaries of MR slices, no organs or tissues exist, so the corresponding signal intensities are zeros, being a rational explanation for padding.

The transition layers of the first two DBs are a combination of a convolutional layer and an average pooling layer. In addition, the transition layers of DB3 and DB4 are a convolutional layer to compress the channels of the outputs. Finally, the transition layer of DB5 is a simple convolutional layer (no BN layers and ReLUs).

The 2×2 average pooling layer embedded in the first two DBs (DB1 and DB2 in Figure 2) to enlarge receptive fields and reduce trained parameters. The number of feature maps is invariant in pooling layers, while the sizes reduce by one half in both width and height.

##### Skip-Connection

Similar to U-Net [22], we propagate low-level (spatial detailed) feature to the decoder network. The feature maps rendered in each layer are compressed to 16 and connected to the corresponding layer in the decoder network (referred to as concatenation in Figure 2). Hence, the segmentation mask each layer in the decoder network produced is based on not only high-level semantic information extracted from all higher-level feature maps but also low-level fine-grained feature from the corresponding level in the decoder network. The principle will be explained in detail in Section 3.2.2.

#### 3.2.2. The Decoder Network

The decoder network is designed to perceive the segmentation maps from the feature maps in different abstract levels. In addition, all the segmentation maps produced will be concatenated and fused to generate the final segmentation $S_{0}$. Up-sampling is adapted to recover the sizes of feature maps to 256×256. $S_{1}$∼$S_{5}$ are segmentation based on only higher-level information. The forward model of the kth level of the decoder network can be formulated as:

(2) $$\begin{array}{cc} \; & t_{k-1}=Conv\left(y_{k-1}\right) \end{array}$$

(3) $$\begin{array}{cc} \; & S_{k}=Sigmoid\circ H\left(S_{k-1}⊕t_{k-1}\right) \end{array}$$

where Conv represents a convolution operator compressing the channels of feature maps to 16. $y_{k-1}$ is the same feature map as it in Equation (1). *H* is a convolutional layer and Sigmoid is a sigmoid function which is defined as:

(4) $$Sigmoid\left(x\right)=\frac{1}{1+e^{-x}}$$

which maps $R^{1}$ to [0, 1]. Hence the value at each pixel of the outputs of sigmoid functions shows the probability that the corresponding pixel within the input image belongs to tumors. Finally, the outputs are binarized to obtain $S_{0}$∼$S_{5}$ and the classification threshold is 0.5.

From Equation (2), we derive that $S_{k}$ only depends on $y_{k-1}$ and $S_{k-1}$. $y_{k-1}$ preserves low-level spatial information extracted by the first *k* DBs. So each level of the decoder network leverages the spatial information through skip-connection architecture.

### 3.3. Multi-Level Integrated Loss Function

Similar to [5], an integrated loss function is defined to realize multi-level and multi-scale supervision. Since $S_{0}$∼$S_{5}$ are segmentation obtained from different abstract levels, we include the difference between $S_{0}$∼$S_{5}$ and ground truth as a part of the defined loss. Furthermore, we involve dice loss as a typical metric of medical image segmentation. Hence, our loss function for one segmentation mask produced can be formulated as:

(5) J(P)=CE(P)+DL(P)

CE is pixel-wised weighted cross entropy (Equation (1) in [7]) loss function, which is defined as:

(6) $$CE\left(P\right)=-\frac{\sum_{i,j}\lambda \omega G_{i,j}\log P_{i,j}+\left(1-G_{i,j}\right)\log\left(1-P_{i,j}\right)}{V}$$

where $G_{i,j}$ is the value of pixel within ground truth (manual Nasopharyngeal Carcinoma segmentation by experienced oncologists). $P_{i,j}$ is that within the predicted segmentation mask. *V* is the area (i.e., the number of pixels) of the ground truth or the prediction. Since the tumor region always occupies a small part of a MR slice, it raises the problem of imbalanced foreground and background, which will lower predictive performance, especially for the minority class (referred to tumor area in the proposed model) ω is a weight to balance minor tumor and major normal organs, which is defined by $\omega =\frac{V-V_{P_{1}}}{V_{P_{1}}}$. $V_{P_{1}}$ is the area of predicted tumors in MR slices (the number of pixels labeled one within the prediction). λ is a coefficient to control the influence of the weight *w*.

DL is generalized weighted dice loss [37]:

(7) $$DL\left(P\right)=1-2\frac{\omega_{1}|P_{1}\cap G_{1}|+\omega_{0}\left|P_{0}\cap G_{0}\right|}{\omega_{1}\left(\right|P_{1}\left|+\right|G_{1}\left|\right)+\omega_{0}\left(\right|P_{0}\left|+\right|G_{0}\left|\right)}$$

where $P_{1}$ is the same as *P* and $P_{0}$ is 1−P. Similarly, $G_{1}$ is the same as *G* and $G_{0}$ is 1−G. The weights $\omega_{1}$ and $\omega_{0}$ are defined as $\frac{1}{|G_{1}|}$ and $\frac{1}{|G_{0}|}$ respectively. As mentioned above, the multi-level losses for $S_{0}$∼$S_{5}$ are to be summed up to enhance segmentation performance (see Section 3.3).

## 4. Experimental Evaluation

To evaluate our model, we conducted preliminary experiments. The model is built up on PyTorch platform. We performed the experiments using a server with four Tesla P100 GPU with 16GB RAM. The hyper-parameters to be tuned include epoch, drop rate, and batch size. In addition, the number of trainable parameters is 0.28 M. The epoch, drop rate, growth rate, and batch size are set to 300, 0.2, 12, and 8 for all the following experiments.

### 4.1. Data Acquisitions

Forty T1-weighted (T1) MR scans have been collected from 30 patients diagnosed with NPC. Thirty of them are selected randomly as the training set, and the other is used for testing. The data are re-sampled to 256×256 with the spatial resolutions fixed to 1 mm using cubic spline interpolation. In addition, all scans have been re-oriented to $RAS^{+}$ coordinate system.

### 4.2. Training Details

We trained the model through the stochastic gradient descent algorithm (SGD). The 40 training scans are divided into a training set composed of 30 composed of 10 scans. The learning rate is 0.0001, and the batch size is 8. Our training steps involve three stages to enhance the performance of DCNet: First, we freeze all the layers in the decoder network, and backward gradient propagation only acts on the training parameters of the encoder network. Then, the layers in the encoder network are frozen, and the decoder network is trained by SGD. Finally, we do not freeze any layers, and the whole model is integrally trained. In each stage, the model is trained for equal one hundred epochs. Concurrently, the learning rate is set to be multiplied by 0.1 if the Dice Similarity Coefficient evaluated on the validation set has not risen for ten epochs.

The average time of learning of one epoch is 141.14 s. In addition, the average time of processing a single MR slice is 1.03 s.

### 4.3. Experimental Results

The proposed model is compared with universal and representative models as for tumor segmentation, including FCN [38], U-Net [22] and U-Net++ [39] as baseline models. The models are mainly evaluated according to Dice Similarity Coefficient (DSC). It is a typical metric of medical image segmentation. It can be formulated as:

(8) $$D\left(P,G\right)=\frac{\left|P\cap G\right|}{\left|P|+|G\right|}$$

where *P* and *G* is the predicted segmentation and ground truth, respectively. |P| represents the number of pixels within the region *P*. The experimental results are summarized in Table 1.

Concurrently, five other widely used metrics are selected for auxiliary evaluation:

**Table 1:** Quantitative segmentation results of DCNet and the baseline models. The learning rate is 0.0001 and the batch size is 8. All models have been trained for 300 epochs on the same training set, and are evaluated on the same testing set in every cross-validation trial. The number of cross-validation trials is 4. ↑ means the higher value is better, while ↓ means the lower value is better. The best results are shown in boldface.

Net	DSC ${}^{1}$ ↑	SEN ↑	SPE ↑	PPV ↑	VOE ↓	RVD ↓
U-Net [22]	0.706	0.583	**0.998**	**0.929**	0.452	0.358
U-Net++ [39]	0.715	0.744	0.995	0.728	0.438	0.332
FCN [38]	0.742	0.732	0.996	0.767	0.405	**0.139**
**DCNet (ours)**	**0.773**	**0.854**	0.995	0.732	**0.363**	**0.321**

${}^{1}$ The *p* value was calculated by performing Mann–Whitney U Test on the main metric DSC. Two-tailed p<0.05 indicates a significant difference. In addition, the *p* values of the paired tests between DCNet and FCN, U-Net++, and U-Net are 0.200, 0.029, and 0.029 respectively. So, our method outperforms U-Net and U-Net++ significantly.

#### 4.3.1. Sensitivity (SEN)

The SEN shows the percentage of tumors detected. It can be formulated as:

(9) $$SEN=\frac{\left|P\cap G\right|}{\left|G\right|}$$

#### 4.3.2. Specificity (SPE)

The SPE shows the percentage of normal region correctly segmented. It can be formulated as:

(10) $$SPE=\frac{|P_{1}-P\cup G|}{|P_{1}-G|}$$

where $P_{1}$ is a three-dimensional tensor with the same size as *P* and *G*, all of which elements labeled 1.

#### 4.3.3. Positive Predictive Value (PPV)

The PPV is the precision of segmentation results. It can be formulated as the following.

(11) $$PPV=\frac{\left|P\cap G\right|}{\left|P\right|}$$

#### 4.3.4. Volumetric Overlap Error (VOE)

The VOE [40] reflects the error rate of segmentation results, which is defined as the following.

(12) $$VOE=1-\frac{\left|V\cap G\right|}{\left|V\cup G\right|}$$

#### 4.3.5. Relative Volume Difference (RVD)

The RVD [40] reflects the difference between segmentation results and Ground Truth. It is defined as the following.

(13) $$RVD=\left|\frac{\left|P|-|G\right|}{\left|G\right|}\right|$$

According to Table 1, in terms of the main metric DSC, our proposed model outperforms any other baseline model with a value of 0.773. In terms of the main metric DSC, a significant improvement of 4.17% on FCN, the baseline model with the best performance with a value of 0.742, can be observed. DCNet also performs top three in terms of all the auxiliary metrics. So, its effectiveness is proven.

**Figure 4:**
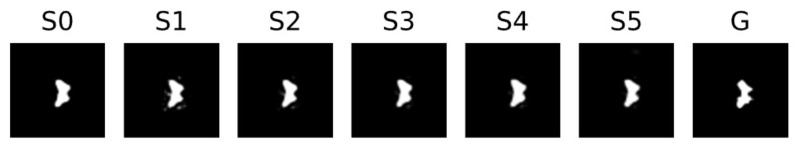
An example of outputs at each level of the decoder network. $S_{i}$ represents the same as that in Figure 2. *G* is ground truth. Please note that the boundaries of $S_{4}$ and $S_{5}$ are smoother than the boundaries of $S_{1}$ and $S_{2}$ caused by the loss of low-level spatial information. However, $S_{1}$ and $S_{2}$ have a few dots mislabeled outside the main tumor region labeled resulting from the lack of context information. In addition, $S_{0}$ fuses them, so its boundary is relatively clear without noticeable mislabeled dots.

To verify the interpretation of the proposed model (see Section 3), Figure 4 illustrates an example of segmentation results produced by different levels of the decoder network. Please note that the outputs at low levels ($S_{1}$, $S_{2}$) preserve more spatial information since the edges of the left half of the results are rougher and fit ground truth better than that of results in higher levels ($S_{4}$, $S_{5}$). However, there are a few dots outside and around the tumor region labeled. We suppose that the mislabeling is caused by a lack of context information, which accords with the interpretation made in Section 3.

**Figure 5:**
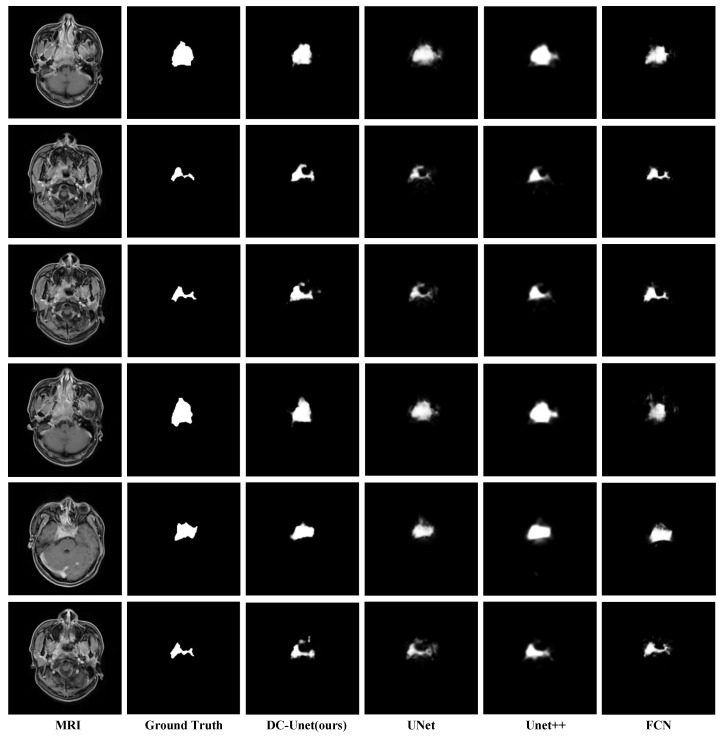
Visual comparison of segmentation results of DCNet and baseline methods. Please note that the segmentation results of our DCNet are more sharper when compared to U-Net and U-Net++. Additionally, the boundaries of the results of DCNet also fit ground truth better than the baseline models.

Figure 5 illustrates a visual comparison of segmentation results of tumors region with different sizes among DCNet and other baseline models. The results show our model performs better than the baseline models and gives a consistently precise result. Hence our model can handle the scale variation problem mentioned in Section 1.

### 4.4. Ablation Analysis

To demonstrate the effectiveness of the network we designed, we conducted four ablation studies to validate the performance of main designed components, including the PPM, the skip-connection architecture, and the integrated loss function. Additionally, we validate that the number of DBs, which is five, is most effective. It keeps a balance between the ability to extract semantic features and the scale of training parameters. The DCNet we proposed is referred to as DCNet-A, and its variants for ablation studies are DCNet-B, DCNet-C, DCNet-D, DCNet-E, DCNet-F, and DCNet-G. All the variants are trained and validated using the same procedure as described in Table 1 Each ablation experiment is performed 3 times and the results are averaged and shown in the following tables and figures.

#### 4.4.1. Effectiveness of Key Modules of DCNet

##### Pyramid Pooling Module

**Figure 6:**
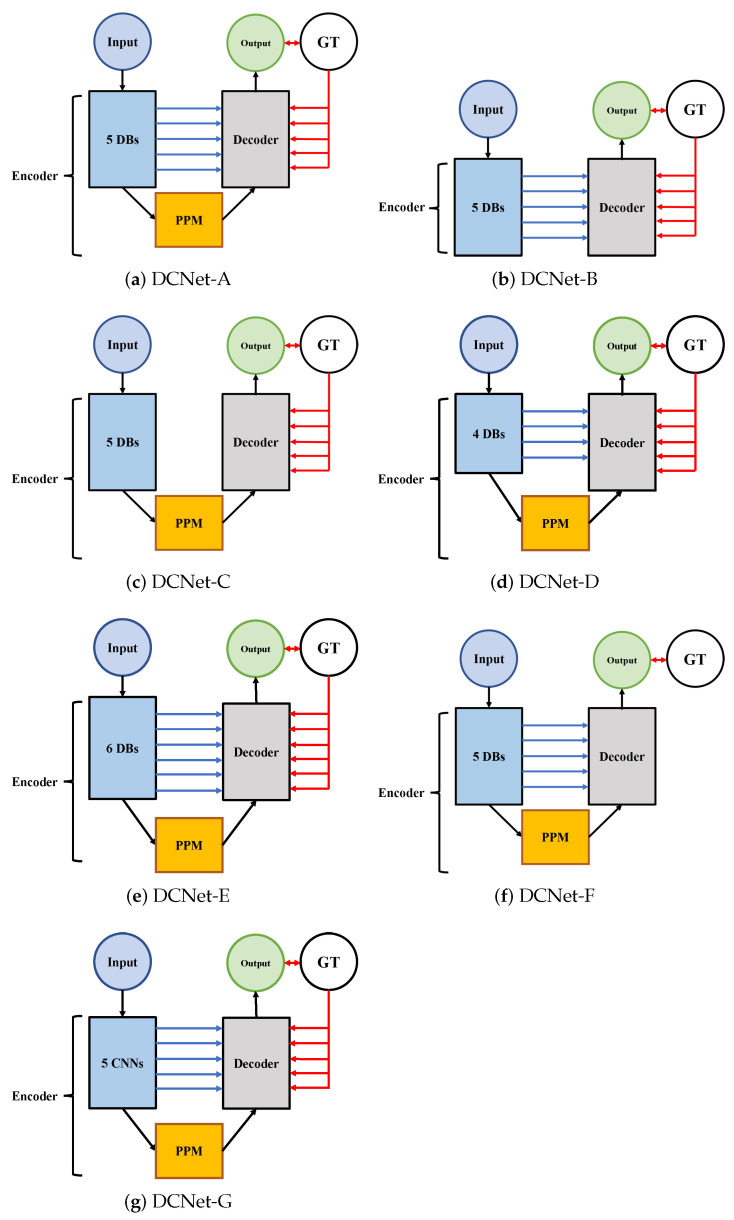
The structures of DCNet and its variants for ablation studies. 5 DBs represent five Dense Blocks and GT meaning ground truth. 5 CNNs means five DBs without dense connection. Blue lines are skipped connections. PPM is the pyramid pooling module. The architecture of the decoder is provided in Section 3.2.2. The models with five red arrows towards decoder have the integrated loss function.

To measure the contribution of PPM, we design a baseline model without PPM: DCNet-B in Table 2. From Table 2, DCNet-A outperforms the baseline model in terms of all the metrics provided. Its architecture is illustrated in Figure 6. The quantitative results in the table show PPM improves the segmentation performance of the model.

##### Skip-Connection Architecture

As mentioned above, we suppose the skip-connection architecture functions as a pathway to convey the low-level spatial feature in the encoder network to the decoder network. Hence it boosts the performance of the model. To verify the argument, we derive a comparative model: DCNet-C in Table 2. Its structure is summarized in Figure 6. From the table, we observe DCNet-A provides the best results in terms of all the metrics. It has a 15.7% improvement in terms of DSC compared to DCNet-C. So, the skip-connection architecture is necessary for boosting the performance of the model.

##### Integrated Loss Function

We have made the argument that the novel loss function plugged in contributes to the improvements of multi-scale GTV detection. To substantiate it, we applied only one loss function (the same formulation as Equation (5)) to evaluate the difference between the segmentation result generated at the highest level of the decoder network $S_{0}$ and the ground truth in the training process. It is referred to as DCNet-F in Figure 6. Table 3 shows integrated loss functions can enhance the performance of segmentation. DCNet-A with an integrated loss function outperforms DCNet-F on the main metric DSC and most of the auxiliary metrics. The only metric where DCNet-F obtains a better result is RVD. Hence, the argument made is strongly proven.

##### The Number of Dense Blocks

We investigate whether five is the optimal value of the number of DBs. So, we design two baseline models: DCNet-D with four DBs and DCNet-E with six DBs. A significant decrease in performance is clearly observed from Table 4. The DSC of DCNet-D with four DBs is 47.6% lower than DCNet-A with five DBs. The DSC of DCNet-E with six DBs is 40.4% lower than DCNet-A. In addition, DCNet-A performs better than the other two comparative models in terms of all the auxiliary metrics except RVD. A direct interpretation is that the depth of DCNet-D with four DBs is not adequate for dealing with large intraclass variation [41] of scale and shape of tumors, while DCNet-E with six DBs is exceedingly deep, causing the gradient vanishing problem. Collectively, five is the optimal choice.

##### Dense Block

**Figure 7:**
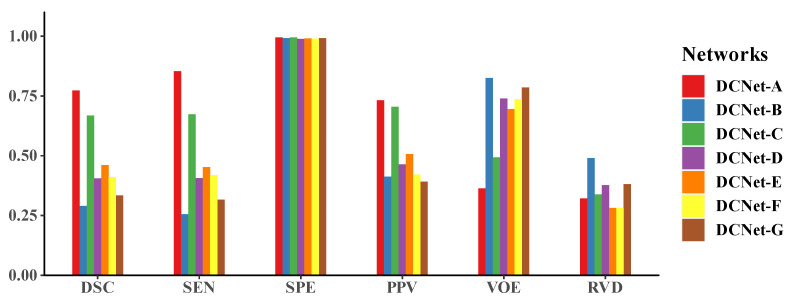
Comparison of the evaluation metrics of DCNet-A and its variants. This is a summary of Table 2, Table 3, Table 4 and Table 5. The results show PPM contributes most to enhancing the performance of DCNet.

To validate the effectiveness of the Dense Blocks, we remove the dense connection in the five DBs (referred to as DCNet-G in Figure 6). The comparative model includes five blocks. Each block is composed of 12 BN + ReLU + Conv layers. In addition, each block produces a feature map with the same number of channels as the outputs of the corresponding DB. The quantitative results are summarized in Figure 7. The comparative model performs significantly worse than DCNet-A in terms of all metrics. The values of DSC, SEN, SPE, and PPV decrease by 53.8%, 63.0%, 0.3%, and 46.4% respectively. In addition, the increase rates of VOE and RVD are 116.5% and 18.7%, respectively. The quantitative results are shown in Table 5.

#### 4.4.2. Comparison of the Influence of Modules

As is illustrated in Figure 7, a comprehensive comparison of the performance of DCNet and its variants is intended to investigate the relative contribution of the designed modules. The figure shows the performance of DCNet-B without PPM is worst. Hence PPM is the vital component of DCNet to boost the performance of segmentation. The modules proposed sorted by contribution, from major to minor, are PPM, DB, the integrated loss function, and the skip-connection architecture, according to the comparison of most metrics. In addition, the number of DBs also has significant influence on the segmentation performance, more than the loss function and connection.

## 5. Conclusions and Future Work

In this paper, a densely connected deep convolutional encoder–decoder network is proposed for Nasopharyngeal Carcinoma segmentation in MR slices. The network is composed of the encoder network and the decoder network for location of tumor regions and boundary-awareness (the ability to detect and label the edges of tumors precisely). In the encoder network, we use densely connected block and pyramid pooling modules to extract high-level semantic features to locate tumors. In addition, the decoder network is designed to obtain masks. Skip-connection architecture is involved and modified to propagate low-level spatial features to the decoder network. Hence it integrates spatial information propagated and semantic information fed by pyramid pooling module. Additionally, masks generated at each level are fused (concatenated and convoluted) to acquire fine-grained segmentation. In terms of training strategy, we also propose an integrated loss function to remedy the gradient vanishing problem by strengthening the gradient signal at each level of the decoder network.

Although our method shows satisfactory results, we suppose there are some limitations and potential improvements. The limitations and future work are summarized as follows:

1. The influence of the value set of the hyper-parameters has not been investigated.
2. The network only processes a single 2D slice at one time. So, we think the network can take 3D image series as inputs by redesigning parts of the network, to speed up the prediction.
3. We consider using transfer learning methods, by training the network on the other data containing other tumors in MR images, to solve the problem of few data.
4. We have noticed Generative Adversarial Network (GAN) is used in web data to resolve the class imbalance problem [42]. In addition, we suppose it is a potential way to resolve the problem of intraclass variation and lack of sufficient data in our research. We consider involving a GAN in the preprocessing stage.
5. We have noticed transformer is used for segmentation tasks for other types of tumors and organs such as MedT [43] and UTNet [44]. These models also outperform baseline models. Therefore, we plan to compare our method to new transformer methods to seek potential modifications.
6. We doubt whether it performs well in cases with other types of tumors, and we plan to investigate in the future.

## Figures and Tables

**Table 2:** Quantitative evaluation metrics of DCNet and its variants. The learning rate is 0.0001, and the batch size is 8. The architecture of the models is illustrated in Figure 6. All the models have been trained for 300 epochs on the same training set and are evaluated on the same testing set. The best results are shown in boldface. The results show DCNet-A outperforms the other comparative models in terms of the majority of all the metrics except SPE. So, the effectiveness of PPM and skip-connection is validated.

Methods	DSC ↑	SEN ↑	SPE ↑	PPV ↑	VOE ↓	RVD ↓
**DCNet-A (original)**	**0.773**	**0.854**	**0.995**	**0.732**	**0.363**	**0.321**
DCNet-B (no PPM)	0.290	0.255	0.992	0.412	0.825	0.490
DCNet-C (no skip-connection)	0.668	0.673	**0.995**	0.705	0.493	0.338

**Table 3:** Quantitative evaluation metrics of DCNet-A with the integrated loss function and DCNet-F with the single loss function. The learning rate is 0.0001, and the batch size is 8. The loss function of DCNet-F is defined as $6J(S_{0})$, while the loss function of DCNet-A is defined as Equation (5). Both models have been trained for 300 epochs on the same training set and are evaluated on the same testing set. The better results are shown in boldface. The results show DCNet-A outperforms the other comparative models in terms of the majority of all the metrics except RVD. Hence the integrated loss function contributes to boosting the performance of DCNet.

Methods	DSC ↑	SEN ↑	SPE ↑	PPV ↑	VOE ↓	RVD ↓
**DCNet-A (integrated loss function)**	**0.773**	**0.854**	**0.995**	**0.732**	**0.363**	0.321
DCNet-F (single loss function)	0.410	0.419	0.990	0.421	0.736	**0.281**

**Table 4:** Quantitative evaluation metrics of DCNet and its variants with different numbers of DBs. The learning rate is 0.0001, and the batch size is 8. The encoder network of DCNet-D has four DBs. The transition layers of the DBs remain the same as the transition layers of the first four DBs in DCNet-A. A DB is added between DB5 and PPM in DCNet-F. The sizes of the outputs of the additional DB remain 64×64, while the number of channels is changed to 140. All models have been trained for 300 epochs on the same training set and are evaluated on the same testing set. The best results are shown in boldface. The results validate the optimal number of DBs is five.

Methods	DSC ↑	SEN ↑	SPE ↑	PPV ↑	VOE ↓	RVD ↓
DCNet-D (Four DBs)	0.405	0.406	0.989	0.464	0.739	0.377
**DCNet-A (Five DBs, original)**	**0.773**	**0.854**	**0.995**	**0.732**	**0.363**	0.321
DCNet-E (Six DBs)	0.461	0.452	0.991	0.507	0.695	**0.282**

**Table 5:** Quantitative evaluation metrics of DCNet-A with dense connection and DCNet-G without dense connection. The learning rate is 0.0001, and the batch size is 8. Both models have been trained for 300 epochs on the same training set and are evaluated on the same testing set. The better results are shown in boldface. The results show DCNet-A outperforms the comparative model in terms of all the metrics. Hence the dense connection contributes to boosting the performance of DCNet.

Methods	DSC ↑	SEN ↑	SPE ↑	PPV ↑	VOE ↓	RVD ↓
**DCNet-A (dense connection)**	**0.773**	**0.854**	**0.995**	**0.732**	**0.363**	**0.321**
DCNet-G (without dense connection)	0.334	0.316	0.992	0.392	0.786	0.381

## Data Availability

The data presented in this study are available on request from the corresponding author. The data are not publicly available due to patient privacy.
